# Type 1 diabetes prediction in autoantibody-positive individuals: performance, time and money matter

**DOI:** 10.1007/s00125-025-06434-2

**Published:** 2025-05-10

**Authors:** Lauric A. Ferrat, Erin L. Templeman, Andrea K. Steck, Hemang M. Parikh, Lu You, Suna Onengut-Gumuscu, Peter A. Gottlieb, Taylor M. Triolo, Stephen S. Rich, Jeffrey Krischer, R. Brett McQueen, Richard A. Oram, Maria J. Redondo, the Type 1 Diabetes TrialNet Study Group

**Affiliations:** 1 Institute of Biomedical and Clinical Science, University of Exeter Medical School, Exeter, UK; 2 Department of Genetic Medicine and Development, University of Geneva, Geneva, Switzerland; 3 Barbara Davis Center for Diabetes, University of Colorado Anschutz Medical Campus, Aurora, CO USA; 4 Health Informatics Institute, Morsani College of Medicine, University of South Florida, Tampa, FL USA; 5 Department of Genome Sciences, University of Virginia, Charlottesville, VA USA; 6 Department of Clinical Pharmacy, University of Colorado Anschutz Medical Campus, Aurora, CO USA; 7 Baylor College of Medicine, Texas Children’s Hospital, Houston, TX USA

**Keywords:** Cost analysis, Genetics, Metabolic, Performance assessment, Predictive model, Type 1 diabetes

## Abstract

**Aims/hypothesis:**

Efficient prediction of clinical type 1 diabetes is important for risk stratification and monitoring of autoantibody-positive individuals. In this study, we compared type 1 diabetes predictive models for predictive performance, cost and participant time needed for testing.

**Methods:**

We developed 1943 predictive models using a Cox model based on a type 1 diabetes genetic risk score (GRS2), autoantibody count and types, BMI, age, self-reported gender and OGTT-derived glucose and C-peptide measures. We trained and validated the models using halves of a dataset comprising autoantibody-positive first-degree relatives of individuals with type 1 diabetes (*n*=3967, 49% female, 14.9 ± 12.1 years of age) from the TrialNet Pathway to Prevention study. The median duration of follow-up was 4.7 years (IQR 2.0–8.1), and 1311 participants developed clinical type 1 diabetes. Models were compared for predictive performances, estimated cost and participant time.

**Results:**

Models that included metabolic measures had best performance, with most exhibiting small performance differences (less than 3% and *p*>0.05). However, the cost and participant time associated with measuring metabolic variables ranged between US$56 and US$293 and 10–165 min, respectively. The predictive model performance had temporal variability, with the highest GRS2 influence and discriminative power being exhibited in the earliest preclinical stages. OGTT-derived metabolic measures had a similar performance to HbA_1c_- or Index_60_-derived models, with an important difference in cost and participant time.

**Conclusions/interpretation:**

Cost–performance model analyses identified trade-offs between cost and performance models, and identified cost-minimising options to tailor risk-screening strategies.

**Graphical Abstract:**

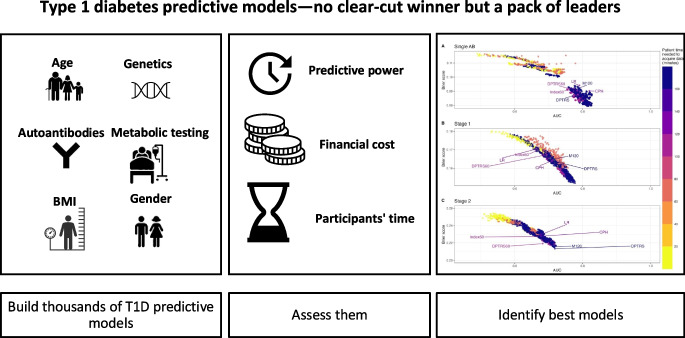

**Supplementary Information:**

The online version contains peer-reviewed but unedited supplementary material available at 10.1007/s00125-025-06434-2.


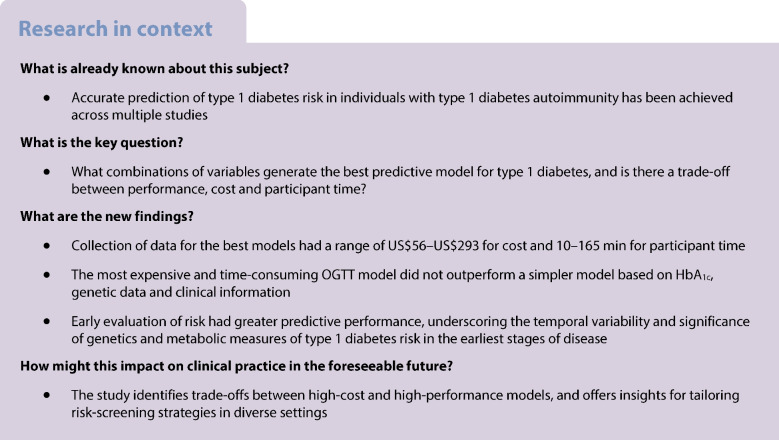


## Introduction

Type 1 diabetes prediction is critical for risk–benefit assessment of therapies to prevent progression to clinical disease [1] and to determine eligibility for prevention trials [2]. In addition, screening and identifying individuals at risk for type 1 diabetes allows monitoring to prevent diabetic ketoacidosis at clinical diagnosis [3, 4], and can provide important insights into disease pathogenesis. Individuals with islet autoimmunity may be categorised as having single autoantibody positivity, stage 1 type 1 diabetes (i.e. multiple autoantibody positivity with normal glucose metabolism), stage 2 type 1 diabetes (i.e. multiple autoantibody positivity and dysglycaemia) or stage 3 (i.e. clinical) type 1 diabetes [5, 6]. These stages have been used to determine monitoring strategies and to understand the pathogenesis of type 1 diabetes. However, there is heterogeneous risk of clinical (stage 3) type 1 diabetes within each of the stages, and enhancing prediction remains a challenge in term of performance, cost and participant burden.

Multiple variables are predictive of type 1 diabetes. These variables are generally grouped into immunological, metabolic, demographic/clinical or genetic categories. Use of combinations of these variables has resulted in promising models [7–9] and scores such as the Diabetes Prevention Trial – type 1 risk score (DPTRS) [10], DPTRS_60_ [11], Model 120 (M_120_) [12], Index_60_ [13], the Cox proportional hazard model (CPH) [14] and the likelihood ratio model (LR) [14]. However, while monitoring all predictors holds the potential to improve prediction, this may inadvertently reduce the feasibility of type 1 diabetes prediction in clinical practice and research. The financial cost and time required from the participant and the physician or clinical researcher may increase with the number of predictors assessed. Thus, a description of the relative cost–benefit associated with the measurement of each variable may help to plan efficient follow-up of at-risk individuals, ensuring a judicious balance between acquisition of comprehensive data and mitigation of undue burden on the participant or the healthcare system. To date, most predictive model studies have focused on finding the model with the best predictive performance, but a comprehensive approach to compare predictive models for performance and cost is lacking.

We compared classical models (DPTRS [10], DPTRS_60_ [11], M_120_ [12], CPH [14] and LR [14]) for type 1 diabetes prediction with predictive models based on a large panel of linear combinations of well-defined predictive variables in terms of three metrics: predictive performance, cost and participant time needed for testing.

We analysed data from participants in Pathway to Prevention, the observational arm of the Type 1 Diabetes TrialNet, an international network funded by the US National Institute of Diabetes and Digestive and Kidney Diseases that aims to prevent type 1 diabetes [2, 15]. This study prospectively follows autoantibody-positive relatives of people with type 1 diabetes for the development of clinical (stage 3) type 1 diabetes. TrialNet participants are at high risk of type 1 diabetes, but whether they progress to clinical type 1 diabetes, and, if they do, the time to onset, remain uncertain, and thus time-efficient, cost-efficient and accurate estimation of their type 1 diabetes risk is needed.

## Methods

### Participants

**Fig. 1:**
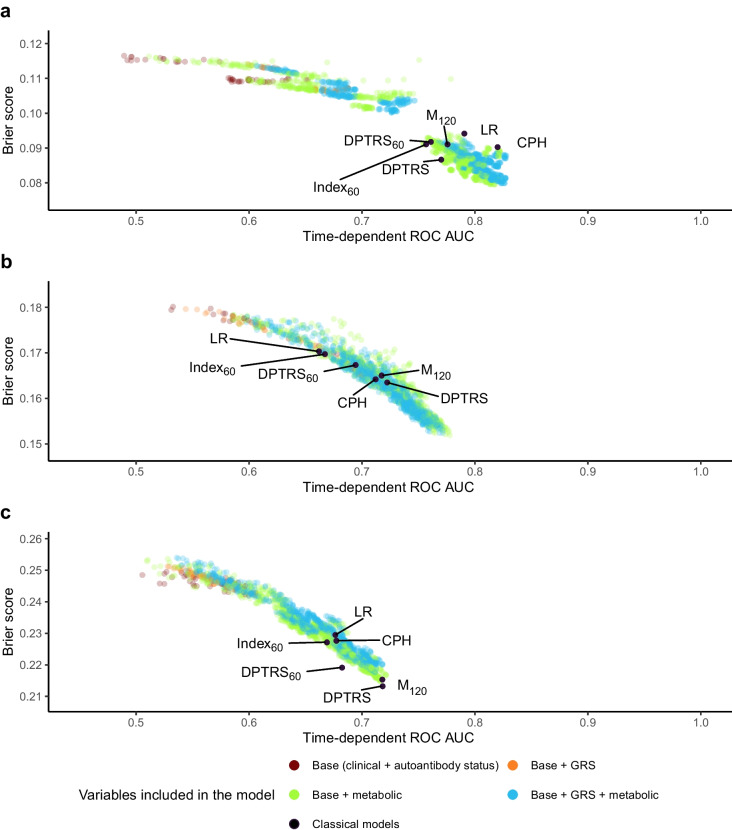
Time-dependent ROC AUC and Brier scores to predict type 1 diabetes at a 3 year horizon for (**a**) single positive autoantibody stage, (**b**) stage 1 diabetes and (**c**) stage 2 diabetes. Each dot represents a CPH model with a different formula. The colour key indicates the class of the variables included in the model. Base models include any combination of BMI *z* score or BMI, age or log_*e*_(age), IA-2A (positive, negative) or autoantibody combination. ‘Metabolic’ includes any combination of variables among AUC glucose, AUC C-peptide, HbA_1c_, Index_60_ C-peptide_30_ or β2 scores. The classical models previously described in the literature are labelled

For the present analysis, we included 4377 TrialNet Pathway to Prevention participants [15, 16] who had been genotyped using the Illumina T1DExomeChip array, had at least one islet autoantibody, and had a baseline OGTT performed less than a year after entering TrialNet. If the participant had a single positive antibody, this was confirmed on two successive tests. We excluded 355 participants who had been diagnosed with diabetes or who had hyperglycaemia, defined as fasting plasma glucose ≥7.0 mmol/l (≥126 mg/dl) 2 h plasma glucose ≥11.1 mmol/l (≥200 mg/dl), or HbA_1c_ ≥48 mmol/mol (≥6.5%) at their baseline OGTT. We further removed 55 participants with incomplete data. A flowchart of the cohort selection is provided in electronic supplementary material (ESM) Fig. 1. The dataset included 49% female participants; 89% of participants were of white European ancestry, and socioeconomic information for the participants was unknown. The participants provided informed consent, and the study was approved by the ethics committee at each site.

### Procedures

#### Study protocol

Participants were initially screened for GADA, IAA and IA-2A. Only a subset of participants were tested for islet cell antibodies and ZnT8 A, and thus these two autoantibodies were not included in this analysis. Participants were monitored using HbA_1c_ measurements and OGTT at 6- or 12-month intervals depending on estimated risk.

#### Diagnosis of type 1 diabetes

During follow-up, type 1 diabetes was diagnosed in participants with hyperglycaemia, i.e. fasting plasma glucose ≥7.0 mmol/l, 2 h plasma glucose ≥11.1 mmol/l after 75 g oral glucose, random plasma glucose ≥11.1 mmol/l, or an HbA_1c_ ≥48 mmol/mol (6.5%). The participants were either symptomatic, or, if asymptomatic, met these thresholds on two separate occasions.

#### Autoantibody assays

GADA, IA-2A and IAA were measured by radioimmunoassay in the TrialNet Core Laboratory at the Barbara Davis Center (Aurora, CO), as previously described [17].

#### Metabolic measures

Participants underwent an OGTT (oral glucose dose 1.75 g/kg, maximum 75 g) after an overnight fast. C-peptide and glucose measurements were performed in the fasting state and 30, 60, 90 and 120 min later.

#### SNP genotyping and computation of the type 1 diabetes genetic risk score

Genotyping was performed using the T1DExomeChip array (Illumina). This is a custom array with more than 90,000 custom SNPs selected from regions of the genome that are robustly associated with autoimmune diseases, the Infinium CoreExome-24 version 1.1 BeadChip. The type 1 diabetes genetic risk score (GRS2) described by Sharp et al [18] includes 67 SNPs, 30 of which were directly genotyped. We imputed 32 SNPs with a median *R*^2^ of 0.997 (min 0.858; max 0.999) using whole-genome sequence data from the NHLBI TOPMed Imputation Server (https://imputation.biodatacatalyst.nhlbi.nih.gov/) and the multi-ethnic reference panel that includes 97,256 reference samples and >308 million genetic variants [19]. We also imputed five SNPs in the HLA region (rs72848653, *R*^2^=0.999; rs9266268, *R*^2^=0.999; rs16899379, *R*^2^=0.998; rs2524277, *R*^2^=0.995; rs9268500, *R*^2^=0.925) using HLA-TAPAS hosted by the Michigan Imputation Server (https://github.com/immunogenomics/HLA-TAPAS/) and the high-resolution HLA reference panel spanning five global populations (*n*=21,546) based on whole-genome sequencing data [20]. Code to generate the HLA interaction part of the GRS2 is freely available online (https://github.com/t2diabetesgenes/t1dgrs2).

### Statistical analysis

We used baseline variables from four categories: clinical/demographic, genetic, metabolic and immunological. In the clinical category, we considered the age (absolute value or natural logarithm) at screening, self-reported gender and BMI (absolute value or age- and gender-adjusted *z* score). To harmonise adult and paediatric BMI values, BMI *z* scores were calculated using previously published tables for children [21] and an age of 20 years for adults, as previously reported [22]. In the immunological category, we either included IA-2A (positive, negative) or autoantibody combination (i.e. GADA, IAA, IA-2A, GADA–IAA, GADA–IA-2A, IAA–IA-2A, GADA–IAA–IA-2A). Metabolic variables included fasting C-peptide, AUC C-peptide, AUC glucose and 30 min C-peptide index (C-peptide_30_). The GRS2 was used to provide an estimated genetic risk for type 1 diabetes. ESM Table 1 summarises the main characteristics of the study participants, both overall and by type 1 diabetes stage at screening.

#### Primary outcome

The primary outcome was time to type 1 diabetes.

#### Modelling

We used two modelling approaches. The first is the Cox proportional hazard (CPH) model [23], which is a classical statistical method to measure the effect of a unit increase of a variable with respect to the hazard rate. The second is the survival random forest approach [24], an ensemble learning method that can capture non-linear effects and interactions but may increase the risk of overfitting.

We trained the model using half of the dataset (participants entering the study during or before January 2013) and validated the model on the second half of the dataset (observations entering the study from February 2013). Only validation results are shown.

To compare the modelling approaches, we used measures of predictive power adapted to the survival setting and able to correctly adjust from censoring, i.e. time-dependent receiver operating characteristic (ROC) AUC [25] and the time-dependent Brier score [26]. The formula for the Brier score is given in ESM Methods. Time-dependent ROC AUC measures the discriminative power of a model to predict an event between the present and a chosen future time horizon; a score of 1 indicates perfect discrimination while a score of 0.5 is equivalent to no discrimination. The Brier score measures the agreement between predicted and observed risk; a value closer to 0 indicates a better performance. The time-dependent ROC AUC and Brier score were calculated for each model at 2-, 3-, 5,- 7- and 10-year horizons. We generated a measure of variable importance to assess the relative predictive power of each variable (see [27] and ESM Methods). The results were computed using R version 4.3.2 with the packages survival [25, 28] and randomForestSRC [29]. *p* values to compare time-dependent ROC AUC were obtained using the approach developed by Blanche et al [25].

#### Generation of type 1 diabetes predictive models

For the variables used in this study, there were 114,688 combinations that may be used to fit a predictive model. To limit the number of combinations of variables, we imposed rules to avoid strong collinearity (such as BMI and BMI *z* score not appearing in the same model). A full list of the rules is presented in ESM Methods. In total, we fitted 1943 combinations of variables using the CPH model and survival random forest model for each stage of progression to type 1 diabetes. We compared the performance of these models to that of DPTRS [10], DPTRS_60_ [11], M_120_ [12], Index_60_ [13], CPH [14] and LR [14]. The formulae for each of these models are given in ESM Methods.

#### Identifying models with global best performance

A Pareto front [30] (see ESM Methods) was used to identify the model with the best trade-offs between competing performance measures (i.e. cost, participant time and predictive performance at 3-year horizon as measured by ROC AUC and Brier score for each stage).

### Estimation of financial cost and participant time for each variable

The cost of acquiring data on specific predictors can vary greatly (see Table 1 and ESM Table 2 for a detailed cost breakdown). Some are easily accessible (e.g. age), while others, such as metabolic variables, may require sequential blood sampling during an OGTT, adding to the cost and time required from the participants and the research or healthcare teams. We followed previous guidelines to estimate the cost of each variable [31–33]. All costs were inflated to their net present value in 2024 US dollars. Direct costs were calculated using US Medicare reimbursement rates as these are a measure of central tendency on the private market and include costs of the test and healthcare provider time. Indirect costs were calculated using wage data from the US Bureau of Labor Statistics, and accounted for the value of each participant’s time, which was calculated as the lost wages of the participant or the parent/guardian who must accompany paediatric participants [33]. The cost of obtaining the GRS2 was estimated at US$20 based on previous literature [34] and the current cost in research studies. The time needed to obtain the predictors that require a medical procedure (such as an OGTT) was estimated as the duration of the procedure plus 45 min for preparation and discharge tasks. For example, a 120 min OGTT test requires 165 min to account for the time needed before the procedure, such as that required to obtain demographic and anthropometric characteristics and establish an i.v. line, and the time needed after the procedure, such as that required to retrieve the line and discharge the participant. The cost and time ranges apply to a single screening visit. When variables are used simultaneously and can be measured together, then we included assay costs for both but only the time of the longer test. For example, HbA_1c_ can be collected during OGTT, and therefore additional time for this was not included. ESM Table 3 provides an impact inventory (a comprehensive economic assessment of a healthcare intervention [35]).

**Table 1:** Cost estimation for the acquisition of variable information

Variables	Physician time (min)	Assistant’s time to record data (min)	Lab test (HCPCS code)^a^	Participant time (min)	Total cost (US$)
AUC glucose	18.75	5	#82951 + 2 #82952	165	104.2
AUC C-peptide	18.75	5	5 #84681	165	166.7
HbA_1c_	5	5	#83036	10	27.1
Index_60_	12.5	5	#82952 + 2 #84681	105	68.8
C-peptide_30_ score	10	5	#82952 + 2 #84681	75	54.8
β2 score	5	5	#82952 + #84681 + #83036	10	34.1
GRS2	5	5	No HCPCS code^b^	10	37.3
Demographic and anthropometric characteristics^c^	5	5	No HCPCS code^d^	10	17.3

Costs are presented in 2024 US dollars

^a^The numbers preceding the HCPCS code indicate the number of repeats

^b^Estimated at US$20

^c^Any combination of age, BMI or BMI *z* score, and gender

^d^Estimated at US$0

HCPCS, Healthcare Common Procedure Coding System: HCPCS level II codes and descriptors are approved and maintained jointly by the alpha-numeric editorial panel (comprising Centers for Medicare & Medicaid Services, America’s Health Insurance Plans, and the Blue Cross Blue Shield Association)

### Interactive figures

To help the reader to compare predictive models of interest, we developed interactive figures that can be found online at https://lauricf.github.io/interactive_suplement.github.io/. The reader can zoom, identify models with selected variables, or highlight a model of interest across multiples stages.

## Results

This analysis included 3967 TrialNet participants (aged 14.9 ± 12.1 years). The median duration of follow-up was 4.7 years (IQR 2.0–8.1) and 1311 (33%) developed type 1 diabetes during the follow-up. The participant characteristics are shown in ESM Table 1 and a flowchart of the cohort selection is shown in ESM Fig. 1.

### Model performance assessment

We found optimal or equivalent model performance when using Cox models compared with the survival random forest approach. The survival random forest results and pairwise comparison are given in ESM Results. When using Cox modelling, at each stage of preclinical type 1 diabetes, a large range of models had similar performance, with only small differences in time-dependent ROC AUC between the best model and all others (see Fig. 1 for the results at a 3-year horizon, ESM Fig. 2 for the results at a 5-year horizon, and interactive figures). Of the 1943 models in single autoantibody-positive participants, 558 (28.7%) had a similar 3-year horizon ROC AUC (i.e. a difference in ROC AUC of less than 0.03 and *p*>0.05) to that of the best model (which included GRS2, autoantibodies, AUC glucose and HbA_1c_). Similar patterns were observed at type 1 diabetes stages 1 and 2, with 367 of the 1943 models and 396 of the 1943 models, respectively, having a difference in 3-year horizon ROC AUC of less than 0.03 and *p*>0.05.

We observed differences in model performance across the various stages of type 1 diabetes. The ability to discriminate individuals who had progressed to stage 3 (clinical) type 1 diabetes at follow-up was highest for participants with confirmed single autoantibody positivity, as evidenced by a maximal 3-year horizon ROC AUC of 0.83 (95% CI 0.77, 0.88). The overall optimal model performance decreased in stage 1 (maximal 3-year horizon ROC AUC at 0.78 [95% CI 0.73, 0.82]) and stage 2 (maximal 3-year horizon ROC AUC at 0.72 [95% CI 0.66, 0.78]).

The models with the best performance were those based on a combination of AUC glucose, HbA_1c_ and Index_60_, but other models based on GRS2 and Index_60_ achieved similar performance (ROC AUC 0.80; *p*>0.05 compared with the best model). For participants in stage 1, optimal model performance was consistently associated with incorporating at least one metabolic variable (e.g. HbA_1c_, AUC glucose and/or AUC C-peptide). Some models based on HbA_1c_ and Index_60_ had similar predictive power (3 year horizon ROC AUC 0.74; not significantly different from the best model). For participants in stage 2, the best models were DPTRS, M_120_ and models based on HbA_1c_, AUC glucose and Index_60_. Some models based on HbA_1c_ and Index_60_ had similar predictive power (3 year horizon ROC AUC=0.71, *p*>0.05 compared with the best model).

Some variables consistently added predictive power, such as glucose AUC, while others only added predictive value at specific stages. The latter is the case for C-peptide AUC, which, on average, did not add to the predictive power (*p*=0.187 for 3 year horizon) in participants with a single positive autoantibody but did add to the predictive power in participants at later stages (*p*<0.001 for 3 year horizon).

Inclusion of GRS2 improved prediction in single autoantibody-positive participants for all prediction horizons, e.g. mean ROC AUC improvement of 0.025 at 3 years (*p*<0.001) and 0.064 at 7 years (*p*<0.001), and assessment of the variable importance measures showed that, for some specific combinations of variable, GRS2 was the leading variable (see ESM Fig. 3). We found that GRS2 did not improve prediction at stages 1 and 2 for ‘short-term’ horizons, such as 3 years (mean ROC AUC improvement <0.002; *p*>0.05) (ESM Fig. 4), but prediction was improved over longer horizons, e.g. mean ROC AUC improvement of 0.028 at the 7-year horizon (*p*<0.001) (see ESM Fig. 5).

Age, the natural logarithm of age, BMI or BMI *z* score generally only marginally improved the performances of the model (*p*>0.05 and/or a small effect size; mean ROC AUC improvement <0.01), but they can, in certain contexts, add to predictive performance (e.g. the BMI *z* score was an important variable in a model with Index_60_ at stage 2; ESM Fig. 6).

Classical models belonged to the cluster with superior performance. At stage 2, DPTRS and M_120_ models had the best predictive performance.

### Cost analysis

**Fig. 2:**
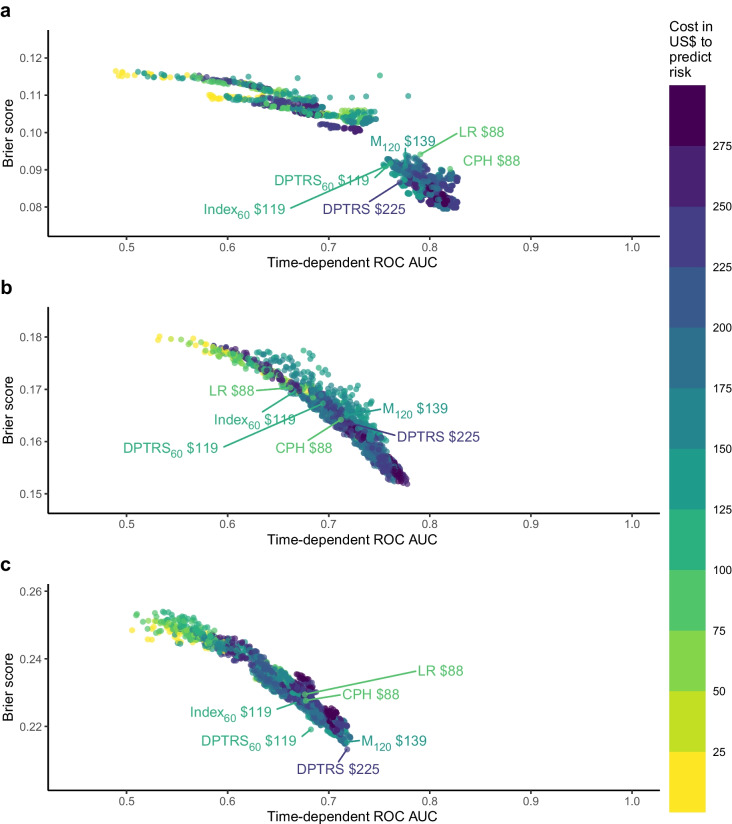
Time-dependent ROC AUC and Brier scores at a 3 year horizon. Each dot represents a CPH model with a different formula. The colour scale indicates the cost of each model. The classical models previously described in the literature are labelled

We observed wide variation in cost for the best-performing models (see Fig. 2 for the performance at the 3 year horizon, ESM Fig. 6 for the performance at the 5-year horizon, and interactive figures. Many high-cost models, characterised by a composite of C-peptide AUC, glucose AUC, additional clinical variables and genetic markers, exhibited good predictive performance across all stages. However, they required a substantial investment (up to US$293) for data collection. The CPH and LR present a cost advantage (US$88) over most predictive models with comparable performance levels. Formulae based on Index_60_ occupied an intermediate position, with mid-level performance and cost (approximately US$125 depending on other variables). Models based on HbA_1c_ and clinical variables outperformed other models based on cost (<US$60).

### Participant time considerations

**Fig. 3:**
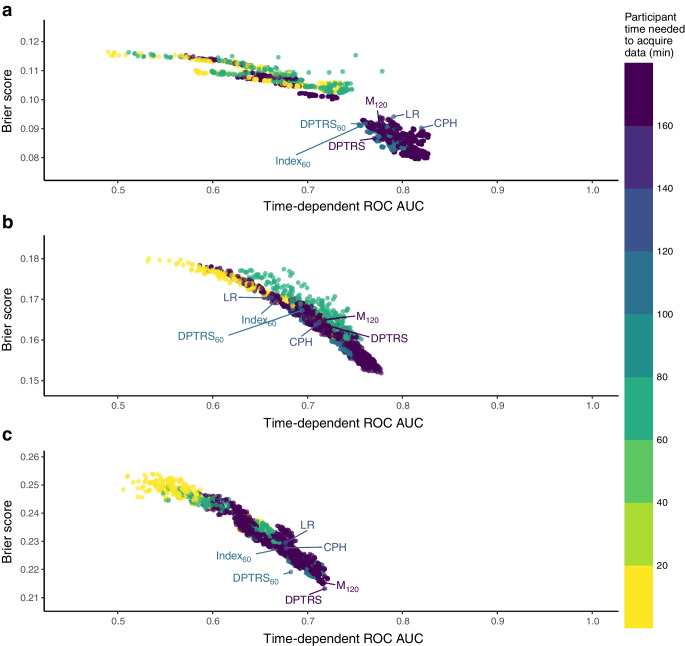
Time-dependent ROC AUC and Brier scores at a 3 year horizon. Each dot represents a CPH model with a different formula. The colour scale indicates the participant time (min) needed to obtain the necessary data for each model. The classical models previously described in the literature are labelled

Given the pivotal role of metabolic variables in driving predictive performance, their inclusion in the model emerged as imperative, and thus it is not possible to achieve good performance without the participant’s engagement that is required for these measures (see Fig. 3 for time performance at 3 year horizon, and ESM Fig. 7 for time performance at 5-year horizon). While models that forego inclusion of metabolic variables shorten the time commitment of the participant, they achieve only moderate performance. However, Index_60_ demands a lesser time investment than other metabolic measures with similarly good predictive performance. Models including the C-peptide_30_ score or the β2 score require less time from the participants compared with Index_60_ and other metabolic measures, but these models have poorer performance.

### Overall best performance

The Pareto front algorithm identified 19 models that were not outperformed by any of the 1943 models, i.e. the combination of their attributes (cost, participant time, predictive performance at a mean 3 year ROC AUC at each stage and mean 3 year Brier score) was not outperformed by any other model for all relevant performance metrics. These models are listed in ESM Table 4, and a list of classical models is provided in ESM Table 5 for comparison. ESM Table 4 indicates that the predictive variables gender, age and HbA_1c_ are over-represented among the best models. The best models may be split into three groups. The first group includes ten models with high performance (less than 5% difference compared with the model with highest performance) but high cost (US$>135) and high participant time (>100 min). In this group, the model IA‑2A + gender + age + (BMI or BMI *z* score) + Index_60_ + HbA_1c_ offers a good trade-off, with a high predictive performance but relative low participant time (105 min) and cost (US$145.95). The second group, comprising five models, offers a good compromise between good predictive performance (less than 10% difference compared with the model with highest performance) and lower cost and/or participant burden. For this set of models, the CPH has the lowest cost (US$88) but requires a high participant time (135 min). In contrast, the model comprising IA‑2A + C-peptide_30_ score + HbA_1c_ has a higher cost (US$119) but requires less time (75 min). The final group, comprising four models, offers lower but relatively good predictive power, and its main advantage is that no OGTT is required, which drastically decreases the cost (<US$100) and participant time (<20 min). In this group, the model IA‑2A + gender + age + HbA_1c_ stands out, with its low cost (US$57) and the second-best predictive performance of the low-cost group.

## Discussion

The relationship between model performance, cost and participant time is an essential consideration for practical implementation that has not been reported on previously. We found that, while some models perform better on specific metrics, none of them stands out as the best in every category. Many models demonstrated commendable performance across all stages, albeit at high cost and high participant burden. In contrast, models such as CPH [14] or LR [14] or models based on Index_60_ [13] achieved comparable performance at lower cost for all stages. This trade-off between performance, cost and participant convenience is pivotal for financially constrained settings where optimal allocation of resources is crucial. Models based on HbA_1c_ offer a good alternative to models based on OGTT measures to predict risk among participants at stages 1 and 2.

Most risk prediction studies of type 1 diabetes to date have focused on whether individual variables are associated with risk of progression, and on overall prediction performance. To our knowledge, our study is the first to formally assess multivariable risk prediction in the context of practical considerations such as financial cost and participant time. Another major strength of this study is use of the TrialNet dataset, which is the largest dataset of individuals at risk for whom data are available on islet autoantibodies, genetics and metabolic measures known to be linked to type 1 diabetes progression.

Our study has several limitations. Most individuals (89%) in our study are of European ancestry with a family history of type 1 diabetes. More work is needed to study type 1 diabetes pathogenesis and progression risk in individuals of non-European ancestry or without a family history. We compared the performance of two modelling approaches, a Cox model and a survival random forest model, and both gave similar results; however, it is possible that another set of models could lead to a different interpretation. To limit the scope of models compared, we did not assess the performance of models that use repeated measures. For example, joint modelling or models based on changes in longitudinal data such as HbA_1c_ [36] or persistence of autoantibody positivity [37] were not considered. Incorporating changes in risk factors over time into future prediction is appealing, and probably offers some advantages, but may increase the complexity of modelling and reduce the ability to provide predictions when individuals are cross-sectionally screened or historical data are not available. Continuous glucose monitoring metrics were not available in this dataset, and thus we could not evaluate their value. It is possible that obtaining increased information on blood glucose levels through use of continuous glucose monitoring may help further refine stages and risk prediction [38]. However, obtaining continuous glucose monitoring measures may come at an increased cost, which also needs to be considered. Another limitation is that cost estimation was based on USA Medicare reimbursement rates, and therefore may not be generalisable to other insurance systems, countries or healthcare systems. However, even if the actual costs are different, our study offers a perspective on the rank order of tests in terms of cost and time required from the participant. Here, we tested the role of variables in prediction and the relationship between them in a study of first-degree relatives who were initially without diabetes and followed prospectively, and it is important to recognise that some results may not apply to other screening settings. Despite the cohort screening selection criterion, i.e. a first-degree relative with type 1 diabetes, we were examining variables that have been shown to be important for type 1 diabetes prediction in many studies. Therefore, our results may have implications for screening in other settings, and the conceptual trade-offs that we examine here are relevant to all efforts to facilitate public health screening for type 1 diabetes.

The GRS2 consistently improved predictive capabilities at the earliest stage (single autoantibody-positive), in alignment with existing genetic susceptibility theories regarding type 1 diabetes onset [18, 39]. This enhancement did not extend uniformly to subsequent stages (stage 1 and stage 2), which partially corroborates our prior finding that GRS2 does not contribute to prediction at higher levels of the DPTRS [39] or in those with multiple autoantibodies [40]. However, when considering a longer-term horizon (5–7 years), GRS2 improved prediction performance to a certain degree (average AUC difference in all models by including GRS2 at stage 1 and stage 2=0.015 and 0.026 respectively, *p*<0.05). These findings illustrate the potential role of genetic factors in shaping the longer-term trajectory of disease progression, while also highlighting the need for nuanced temporal considerations in predictive modelling. As disease-modifying interventions in preclinical stages become the standard of therapy, our study may help to clarify how to practically implement population screening and monitoring in ‘real world’ public health settings. Our results support the use of genetic risk as measured by a GRS2 in the prediction of type 1 diabetes in individuals with a single positive islet autoantibody. However, the weak added predictive value once multiple autoantibodies develop (e.g. stages 1 and 2), even at long-term horizons, does not guarantee the cost-effectiveness of use of GRS2 and requires more investigation. However, obtaining information on genetics requires less time from the participant and the research team than obtaining OGTT-derived metabolic measures. In addition, genetics do not change, and thus can be used repeatedly to reassess the risk for an individual, whereas use of metabolic markers requires the participant to return for each new evaluation.

The good performance obtained with OGTT-derived metabolic variables emerged as a consistent theme in this study and confirms the results of previous studies [41, 42]. Surprisingly, models based on HbA_1c_ were able to attain consistently high predictive performances in all areas assessed at stage 1 and 2, suggesting the possibility that HbA_1c_ could replace the use of OGTT for these stages. Models incorporating at least one metabolic variable consistently outperformed their counterparts that lacked such inclusion (Fig. 1). We observed this most strongly when looking at short-term horizons, and this is understandable given that the diagnosis of clinical, stage 3 type 1 diabetes uses the same metabolic variables. The consistent pattern of inclusion of metabolic variables improving prediction highlights the interaction between the development of autoimmunity and metabolism that is probably taking place even in early disease pathogenesis [43].

Accurate prediction tools are crucial for identifying at-risk individuals and implementing interventions to delay the onset of clinical type 1 diabetes. However, their use in clinical practice remains restricted. While this study compared predictive models across several metrics, it did not evaluate how to use a specific model (e.g. decide which threshold to use for an acceptable sensitivity and specificity) and nor did it make the models accessible in predictive software that complies with US Food and Drug Administration and/or European Union regulations for medical device software. Finally, this study is not a comprehensive cost-effectiveness evaluation. The financial costs, time considerations and predictive performance were assessed independently, lacking an integrating approach in the analysis, which limited readiness for use in decision-making. Addressing these challenges is crucial for enhancing the effectiveness and accessibility of prediction tools for type 1 diabetes screening.

## Supplementary Information

Below is the link to the electronic supplementary material.

ESM (PDF 1632 KB)

## Data Availability

The datasets generated during and/or analysed in the current study are available from the corresponding author upon reasonable request. Interactive figures can be found online at https://lauricf.github.io/interactive_suplement.github.io/.

## Abbreviations

CPH: Cox proportional hazard model
DPTRS: Diabetes Prevention Trial – type 1 risk score
GRS2: Type 1 diabetes genetic risk score
LR: Likelihood ratio model
M_120_: Model 120
ROC: Receiver operating characteristic

## Code availability

The R code and the predictive model parameters that belong to the Pareto front are available at https://github.com/LauricF/ptm_performance.git. Code to generate the HLA interaction part of the GRS2 is freely available online (https://github.com/t2diabetesgenes/t1dgrs2).
