# Supplementary material for: Type 1 diabetes prediction in autoantibody-positive individuals: performance, time and money matter
Source: Diabetologia. 2025 May 10;68(8):1709–20. doi: 10.1007/s00125-025-06434-2 (PMC12245984; doi:10.1007/s00125-025-06434-2)
Supplement: Supplementary file 1 — ESM (PDF 1632 KB) [file 125_2025_6434_MOESM1_ESM.pdf]

Electronic Supplementary Material (ESM)

Table of Contents

Electronic Supplementary Material (ESM) 1
ESM Methods *1*
Time-dependent Brier Score 1
Rules on selection of variables to generate plausible models 1
Formulas for T1D risk scores compared in this study 2
Relative importance for each variable *2*
ESM Results *2*
Comparison of Cox proportional hazard model and survival random forest 3
ESM Tables *4*
ESM Figures *11*
Type 1 Diabetes TrialNet Study Group *19*

ESM Methods

Time-dependent Brier Score

The formula for this score is:

$$Brier_{(t)} = \frac{1}{N}\sum_{i=1}^{N} w_{it}\left[\underbrace{\{I(y_{it} = 0)(y_{it} - \hat{p}_{it})^2\}}_{\text{non-events}} + \underbrace{\left\{I(y_{it} = 1)\left(y_{it} - (1 - \hat{p}_{it})\right)^2\right\}}_{\text{events}}\right]$$

The survival function estimate, $\hat{p}_{it}$, is the probability corresponding to non-events at time *t*.
y_{it} is equal to 1 if the event happened for individual *i* before time *t*, 0 otherwise.
If there has not been an event at the current evaluation time, The best model should estimate the survival probability near one. For observations that are events, the probability estimate is just one minus the survivor estimate. To account for censoring, each observation is weighted with w_{it}.

Rules on selection of variables to generate plausible models

- The **IA2A** variable and the **autoantibody group** variable cannot appear in the same model,
- **Age** and **logarithm of age** cannot appear in the same model,
- **BMI** and **z-BMI** cannot appear in the same model,
- Two of the following variables cannot appear in the same model: **Index$_{60}$**, **AUC C-peptide**, **C-peptide$_{30}$ Index** or **β2-score.**

Formulas for T1D risk scores compared in this study

$$DPTRS = (1.569 \times \log(BMI) + 0.00813 \times (G_{30} + G_{60} + G_{90} + G_{120}) - 0.0848 \times (Cpep_{30} + Cpep_{60} + Cpep_{90} + Cpep_{120}) + 0.476 \times \log(Cpep_f) - 0.056 \times age$$

$$DPTRS_{60} = 1.364 \times \log(BMI) + \big(0.019 \times (G_{60}) - 0.311 \times (Cpep_{60}) + 0.465 \times \log\big(Cpep_f\big)\big) - (0.065 \times age)$$

$$M_{120} = 0.448 \times Male + 0.631 \times IA - 2A - 0.0302 \times age + 0.0605 \times BMI + 1.380 \times HbA_{1c} + 0.0265 \times G_{120} - 0.191 \times Cpep_{120}$$

$$CPH = (HbA1c - 5.233) \times 1.125 + (G_{90} - 107.6) \times 0.0195 + (IA2 - 1.27) \times 0.662)$$

$$LR = 2.048 \times HbA_{1c} + 0.034 \times G_{90} + 0.006 \times IA2$$

$$Index_{60} = 0.395 \times log\big(Cpep_f\big) + 00.0165 \times G_{60} - 0.3644 \times Cpep_{60}$$

$$Index\ C - peptide_{30} = \frac{Cpep_{30} - Cpep_f}{G_{30} - G_f}$$

$$\beta 2\text{score} = \frac{\sqrt{Cpep_f}}{G_f\ HbA_{1c}}$$

G_t indicates Glucose level at time *t*, $Cpep_t$ indicates C-peptide level at time *t*, *f* indicates values at fasting time. HbA1c is measured in %, Glucose (mg/dl), C-peptide (ng/ml).

Relative importance for each variable

The partial effect of each variable was quantified by computing the proportion of explainable log-likelihood explained by each variable, i.e. the Wald χ^2 statistics minus the degrees of freedom (Harrell F. Regression Modeling Strategies. Springer; 2015) between the full model and the model without the variables of interest.
The confidence intervals were generated by running bootstrap a thousand time. A strong risk factor will contribute more, as compared with less a strong risk factor, to the predictive ability of the model.

Pareto front

In many cases, improving one outcome (like cost) might worsen another other (decrease predictive performance), and vice versa. The Pareto front is a concept that helps to understand the best possible trade-offs between competing outcomes.

Key Points of the Pareto Front

- Dominated Solutions: These are predictive models where there is another predictive model that is better in all outcomes of interest. For example, if model A is more costly, require more time than the patient and has less prediction performance than model B, then model A is "dominated" by model B.
- Non-Dominated Solutions (Pareto Optimal): These are models where no other model is better in all outcomes. Essentially, there are no other model that can improve one outcome without worsening at least another. These points form the Pareto front.

The Pareto front was computed with the algorithm developed by Borzsony et al. (https://ieeexplore.ieee.org/document/914855) to identify the set of models which had the best trade-offs between competitive performance measures (cost, participant’s time, predictive performance at 3-year horizon as measured by ROC AUC and Brier score for each stage).

ESM Results

Comparison of Cox proportional hazard model and survival random forest

To determine if Survival random forest models had better performances than Cox proportional hazard models, we did a pairwise comparison by formula and by stages of the time dependent ROC AUC at a 3-year horizon, Pvalue were obtained using the approach developed by Blanche et al (2013). Stat Med 32(30):5381–5397.

In single autoantibody positivity, Cox proportional hazard modeling has better performances ROC AUC at a 3-year horizon in 85 formulas. There was no significative difference for 1590 formulas and survival random forest has better performances in 277 formulas. Further analysis showed that survival random forest only outperformed Cox model when the variable C-peptide$_{30}$ Index is present in the formula (276/277). A post hoc analysis revealed extreme outliers in the C-peptide$_{30}$ Index variable, see ESM figure 8. These outliers negatively impacted the performance of the Cox model but had little effect on the performance of survival random forests, which are inherently less sensitive to outliers.

In stage 1, Cox has better performances in 218 formulas. There was no significative difference for 1590 formulas and survival random forest has better performances in 144 formulas.

In stage 2, Cox has better performances in 115 formulas. There was no significative difference for 1590 formulas and survival random forest has better performances in 247 formulas. As in single autoantibody positivity, further analysis showed that survival random forest only outperformed Cox model when the variable C-peptide$_{30}$ Index is present in the formula (198/247).

Conclusion: Except when C-peptide$_{30}$ Index is present the performance of Cox model is equal or better than survival random forest.

ESM Tables

	Single autoantibody		Stage 1		Stage 2		Overall	
	T1D (*N*=369)	T1D free (*N*=1638)	T1D (*N* =528)	T1D free (*N* =823)	T1D (*N* =414)	T1D free (*N* =195)	T1D (*N* =1311)	T1D free (*N* =2656)
**Gender**								
Female	168 (45.5%)	908 (55.4%)	251 (47.5%)	344 (41.8%)	197 (47.6%)	89 (45.6%)	616 (47.0%)	1341 (50.5%)
Male	201 (54.5%)	730 (44.6%)	277 (52.5%)	479 (58.2%)	217 (52.4%)	106 (54.4%)	695 (53.0%)	1315 (49.5%)
**Age**								
Mean (SD)	14.7 (12.5)	19.2 (13.7)	8.67 (6.50)	12.8 (9.54)	11.8 (9.67)	13.3 (10.6)	11.3 (9.80)	16.8 (12.7)
Median [Min, Max]	10.0 [1.00, 45.0]	13.0 [1.00, 51.0]	7.00 [1.00, 42.0]	10.0 [1.00, 51.0]	9.00 [1.00, 45.0]	11.0 [1.00, 45.0]	8.00 [1.00, 45.0]	12.0 [1.00, 51.0]
**GRS2**								
Mean (SD)	13.8 (1.72)	12.4 (2.33)	13.9 (1.77)	13.4 (1.92)	14.1 (1.61)	13.7 (1.77)	13.9 (1.71)	12.8 (2.23)
Median [Min, Max]	14.0 [6.94, 17.7]	12.7 [4.40, 18.3]	14.0 [8.29, 18.5]	13.5 [5.84, 18.5]	14.1 [8.35, 18.5]	13.9 [8.08, 17.6]	14.0 [6.94, 18.5]	13.1 [4.40, 18.5]
**IA2A**								
Positive	76 (20.6%)	87 (5.3%)	410 (77.7%)	505 (61.4%)	345 (83.3%)	133 (68.2%)	831 (63.4%)	725 (27.3%)
Negative	293 (79.4%)	1551 (94.7%)	118 (22.3%)	318 (38.6%)	69 (16.7%)	62 (31.8%)	480 (36.6%)	1931 (72.7%)
**BMI**								
Mean (SD)	20.8 (6.67)	22.3 (6.75)	18.4 (4.40)	19.9 (5.51)	19.8 (5.57)	20.2 (5.97)	19.5 (5.57)	21.4 (6.43)
Median [Min, Max]	18.0 [12.9, 54.6]	21.0 [8.70, 56.1]	17.1 [12.0, 42.6]	18.4 [8.24, 53.6]	18.0 [12.4, 42.1]	17.7 [12.6, 43.7]	17.5 [12.0, 54.6]	19.8 [8.24, 56.1]
**AUC C-peptide (nm/L)**								
Mean (SD)	2.74 (1.41)	3.37 (1.56)	2.28 (1.06)	2.85 (1.24)	2.53 (1.26)	3.16 (1.44)	2.49 (1.24)	3.20 (1.48)
Median [Min, Max]	2.36 [0.625, 9.36]	3.09 [0.401, 16.8]	2.03 [0.569, 9.09]	2.63 [0.407, 8.02]	2.26 [0.252, 9.42]	2.99 [0.728, 10.2]	2.20 [0.252, 9.42]	2.94 [0.401, 16.8]
**AUC glucose (mg/dL)**								
Mean (SD)	13.1 (2.92)	11.2 (2.05)	11.3 (1.49)	10.7 (1.48)	15.2 (2.31)	13.8 (1.83)	13.0 (2.76)	11.3 (2.02)

Median [Min, Max]	12.7 [7.43, 24.7]	11.0 [6.37, 21.1]	11.2 [7.63, 15.2]	10.7 [4.77, 14.3]	14.8 [9.89, 25.6]	13.7 [8.99, 20.2]	12.7 [7.43, 25.6]	11.0 [4.77, 21.1]
**$Index_{60}$**								
Mean (SD)	0.693 (1.14)	-0.328 (1.13)	0.512 (0.847)	-0.0943 (0.968)	1.39 (0.995)	0.570 (1.08)	0.841 (1.05)	-0.190 (1.11)
Median [Min, Max]	0.775 [-4.28, 4.55]	-0.201 [-8.52, 2.67]	0.606 [-5.10, 2.37]	0.0395 [-4.96, 2.14]	1.50 [-4.19, 3.95]	0.676 [-4.03, 3.09]	0.908 [-5.10, 4.55]	-0.0623 [-8.52, 3.09]
**C-$peptide_{30}$** Index								
Mean (SD)	0.0535 (0.0414)	0.106 (0.357)	0.0561 (0.0541)	0.0907 (0.203)	0.0384 (0.0556)	0.0538 (0.0671)	0.0498 (0.0519)	0.0972 (0.303)
Median [Min, Max]	0.0430 [-0.0144, 0.337]	0.0780 [-4.17, 12.1]	0.0453 [-0.0561, 0.910]	0.0695 [-0.960, 5.25]	0.0293 [-0.0500, 1.00]	0.0433 [-0.319, 0.734]	0.0389 [-0.0561, 1.00]	0.0725 [-4.17, 12.1]
**Beta2 score**								
Mean (SD)	2.50 (0.615)	2.79 (0.664)	2.42 (0.560)	2.65 (0.620)	2.35 (0.616)	2.51 (0.666)	2.42 (0.596)	2.72 (0.656)
Median [Min, Max]	2.42 [0.871, 5.51]	2.73 [0.633, 5.92]	2.33 [1.31, 4.86]	2.57 [1.12, 4.79]	2.27 [1.18, 4.87]	2.48 [1.25, 5.00]	2.34 [0.871, 5.51]	2.67 [0.633, 5.92]
HbA_{1c} **(mmol/mol)**								
Mean (SD)	33.3 (4.06)	32.0 (3.43)	32.1 (3.33)	31.7 (3.21)	34.3 (4.21)	32.6 (3.47)	33.1 (3.94)	31.9 (3.37)
Median [Min, Max]	33.3 [21.3, 53.0]	32.2 [18.0, 54.1]	32.2 [22.4, 45.4]	32.2 [21.3, 46.5]	34.4 [18.0, 59.6]	32.2 [24.6, 51.9]	33.3 [18.0, 59.6]	32.2 [18.0, 54.1]
HbA_{1c}**(%)**								
Mean (SD)	5.20 (0.371)	5.08 (0.314)	5.09 (0.304)	5.05 (0.293)	5.29 (0.385)	5.13 (0.317)	5.18 (0.361)	5.07 (0.308)
Median [Min, Max]	5.20 [4.10, 7.00]	5.10 [3.80, 7.10]	5.10 [4.20, 6.30]	5.10 [4.10, 6.40]	5.30 [3.80, 7.60]	5.10 [4.40, 6.90]	5.20 [3.80, 7.60]	5.10 [3.80, 7.10]

ESM Table 1. Characteristic of the cohorts by stages (single autoantibody positivity, stage 1 [multiple autoantibodies without dysglycemia] and stage 2 [multiple autoantibodies with dysglycemia]) and overall.

Cost categories	Unit cost	Source
	DIRECT COSTS	
Physician time	$66.08/visit	CMS, HCPCS code # 99213
Assistant time	$19.84/h	Bureau of Labor Statistics
Oral Glucose Tolerance Test (OGTT); three specimens (includes glucose)	$12.87	Medicare, HCPCS code # 82951
Blood glucose, each additional beyond 3 specimens in OGTT	$3.92	Medicare, HCPCS code # 82952
Hemoglobin A1C	$9.71	Medicare, HCPCS code # 83036
C-peptide	$20.81	Medicare, HCPCS # 84681
	INDIRECT COSTS	
Patient/participant's time	$14.88/h	half the mean hourly wage in 2022, Bureau of Labor Statistics adjusted to inflation as of 2024
Travel cost	$12.40 / average trip	Zhang et al. (2003) adjusted to inflation as of 2024

ESM Table 2. Cost estimation of each unit included in acquisition of variable information.

Sector	Type of Impact<br>(Add additional domains, as relevant)	Notes on Sources (if quantified),<br>Likely Magnitude & Impact (if not)
Medical Costs	Paid by third-party payers/<br>Paid by patients out-of-pocket	Direct costs were calculated using Medicare reimbursement rates as it is a measure of central tendency on the private market and included costs of the test and healthcare provider time we used Medicare as it cost diagnostic laboratory tests (CDLTs) based off the weighted median of private payor rates (fee schedule)<br>Large impact
Health-Related Costs	Patient time costs + travel	Indirect costs were calculated using wage data from the US Bureau of Labor Statistics and accounted for the value of the participants' time, which was calculated as the lost wages of the participant or the parent/guardian who must accompany pediatric participants<br>Small impact

ESM Table 3. Impact inventory table adapted from Sanders et al. 2016 JAMA

Model	AUC_{S0}	AUC_{S1}	AUC_{S2}	$Brier_{S0}$	$Brier_{S1}$	$Brier_{S2}$	Cost	Time
GRS2 + AB-group + gender + age + BMI + $Index_{60}$ + AUC-glucose + Hba1c	0.82	0.76	0.72	0.08	0.15	0.22	231.27	165
IA-2A + gender + age + $Index_{60}$ + AUC-glucose + Hba1c	0.81	0.76	0.72	0.08	0.15	0.21	193.92	165
GRS2 + AB-group + gender + age + AUC-glucose + Hba1c	0.82	0.76	0.69	0.08	0.15	0.22	189.65	165
GRS2 + IA-2A + gender + age + BMI + $Index_{60}$ + Hba1c	0.81	0.74	0.72	0.08	0.16	0.22	183.3	105
IA-2A + gender + age + AUC-glucose + Hba1c	0.81	0.76	0.69	0.09	0.15	0.22	152.3	165
IA-2A + gender + age + $Index_{60}$ + AUC-glucose + z-BMI	0.81	0.76	0.7	0.08	0.15	0.22	184.21	165
IA-2A + gender + age + BMI + $Index_{60}$ + Hba1c	0.79	0.75	0.72	0.08	0.16	0.22	145.95	105
IA-2A + gender + age + $Index_{60}$ + z-BMI + Hba1c	0.79	0.75	0.71	0.08	0.16	0.22	145.95	105
IA-2A + gender + age + BMI + $Index_{60}$	0.79	0.74	0.7	0.08	0.16	0.22	136.24	105
IA-2A + gender + age + $Index_{60}$ + z-BMI	0.78	0.74	0.69	0.08	0.16	0.22	136.24	105
CPH + GRS2	0.83	0.71	0.68	0.09	0.16	0.23	125.17	135
CPH	0.82	0.71	0.68	0.09	0.16	0.23	87.82	135
$DPTRS_{60}$ + Hba1c	0.77	0.71	0.71	0.09	0.16	0.22	128.61	105
IA-2A + gender + age + C-peptide-index-30 + Hba1c	0.72	0.75	0.67	0.1	0.16	0.23	136.05	75
$DPTRS_{60}$	0.76	0.69	0.68	0.09	0.17	0.22	118.9	105
GRS2 + AB-group + gender + age + Hba1c	0.73	0.73	0.66	0.1	0.16	0.23	94.16	10
IA-2A + gender + age + Hba1c	0.72	0.74	0.67	0.1	0.16	0.23	56.81	10
GRS2 + AB-group + gender + age + BMI	0.68	0.7	0.59	0.11	0.17	0.24	54.7	10
AB-group + gender + age + BMI	0.62	0.71	0.61	0.11	0.17	0.24	17.35	10

Rank
Best model
Worst model

ESM Table 4. Performance of predictive models belonging to the Pareto front across multiple metrics to predict risk at a 3-year horizon. The colour legend indicates for each metric the rank of the model compared to the other models - white indicates that the model was for a given metric among the worst and blue indicates that the model was among the best. Black lines have been added to separate the different clusters.

	AUC_{S0}	AUC_{S1}	AUC_{S2}	$Brier_{S0}$	$Brier_{S1}$	$Brier_{S2}$	Cost	Time
DPTRS + GRS2 + Hba1c	0.8	0.73	0.73	0.08	0.16	0.21	272.21	165
DPTRS	0.77	0.72	0.72	0.09	0.16	0.21	225.15	165
M_{120}	0.78	0.72	0.72	0.09	0.16	0.22	139.2	165
$Index_{60}$ + Hba1c	0.77	0.68	0.7	0.09	0.17	0.22	128.61	105
$DPTRS_{60}$	0.76	0.69	0.68	0.09	0.17	0.22	118.9	105
LR	0.79	0.66	0.68	0.09	0.17	0.23	87.82	135
CPH	0.82	0.71	0.68	0.09	0.16	0.23	87.82	135
IA2 + Sex + age + Hba1c	0.72	0.74	0.67	0.1	0.16	0.23	56.81	10
IA2 + Sex + age + z-BMI	0.61	0.71	0.6	0.11	0.17	0.24	17.35	10

Rank
Best model
Worst model

ESM Table 5. Performance of predictive models of interest across multiple metrics to predict risk at a 3-year horizon. The colour legend indicates for each metric the rank of the model compared to the other models - white indicates that the model was for a given metric among the worst and blue indicates that the model was among the best.

ESM Figures

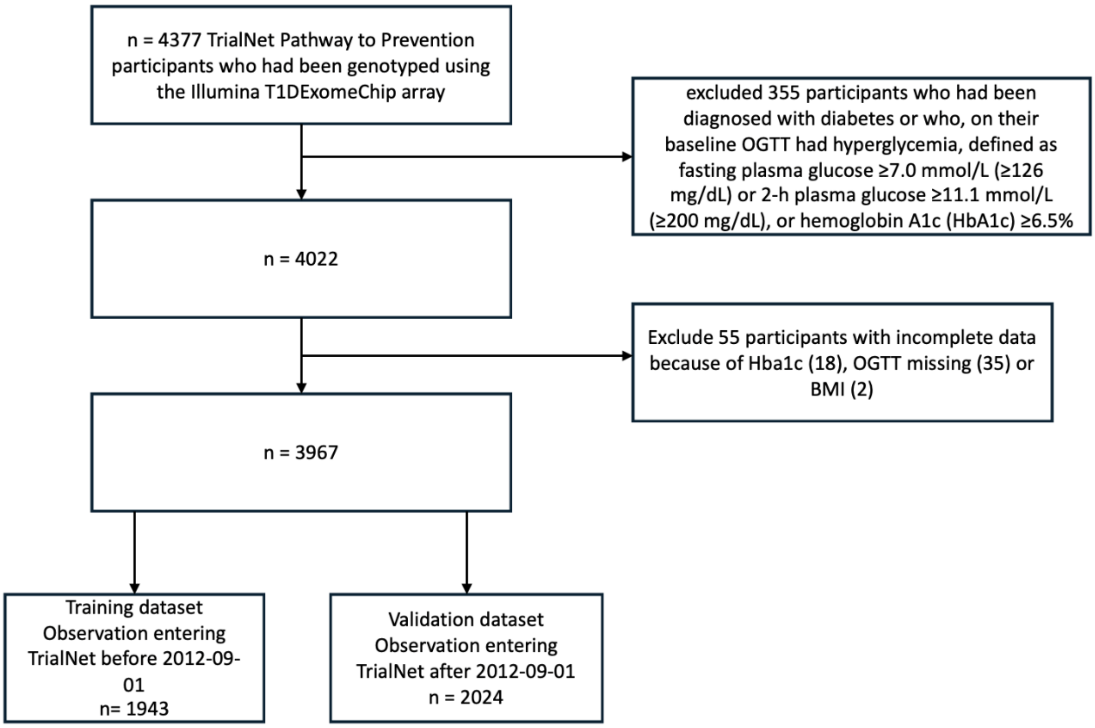

ESM Figure 1. Flowchart of cohort selections and split sample approach

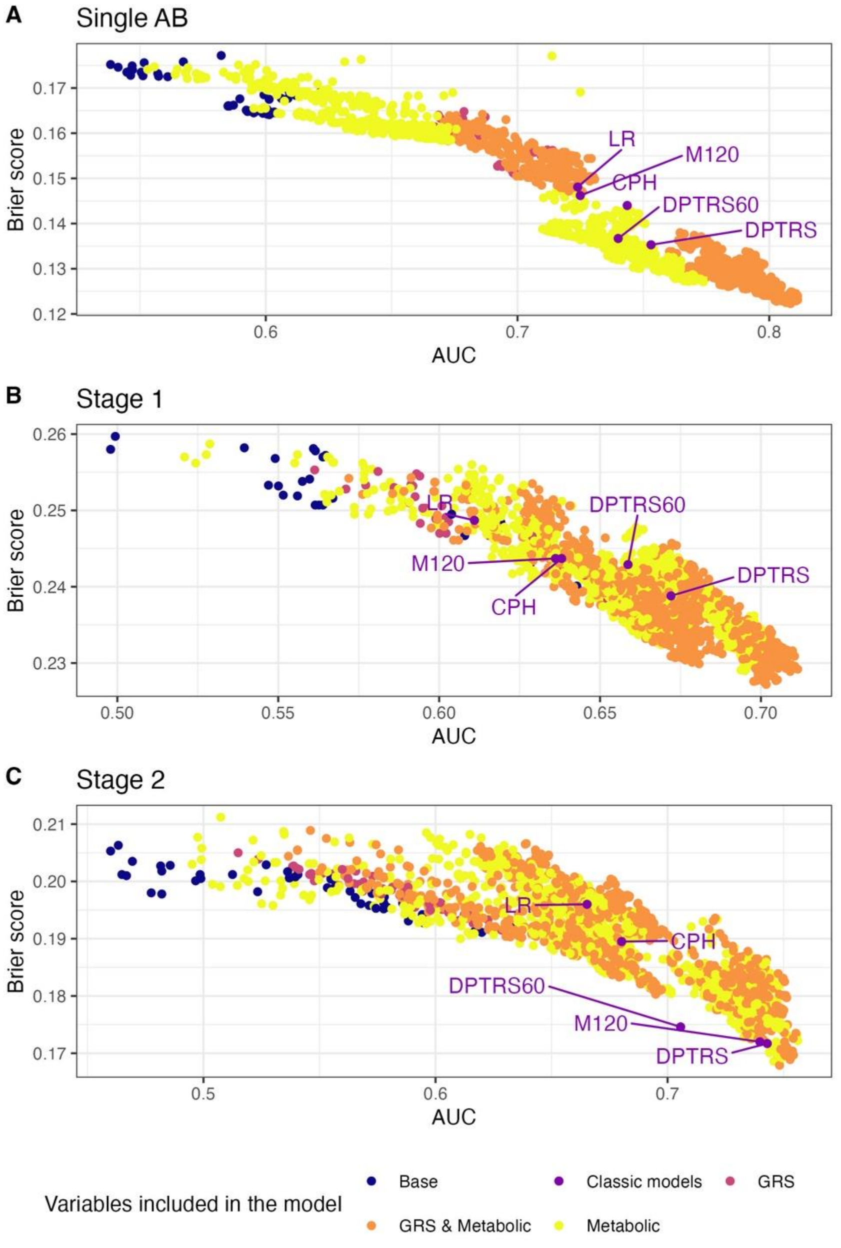

ESM Figure 2: Time dependent ROC AUC and Brier score at a 5-year horizon. Each dot represents a Cox proportional hazard model with a different formula. The color scale indicates the class of the variables included in the model. Classic model previously described in the literature are labelled.

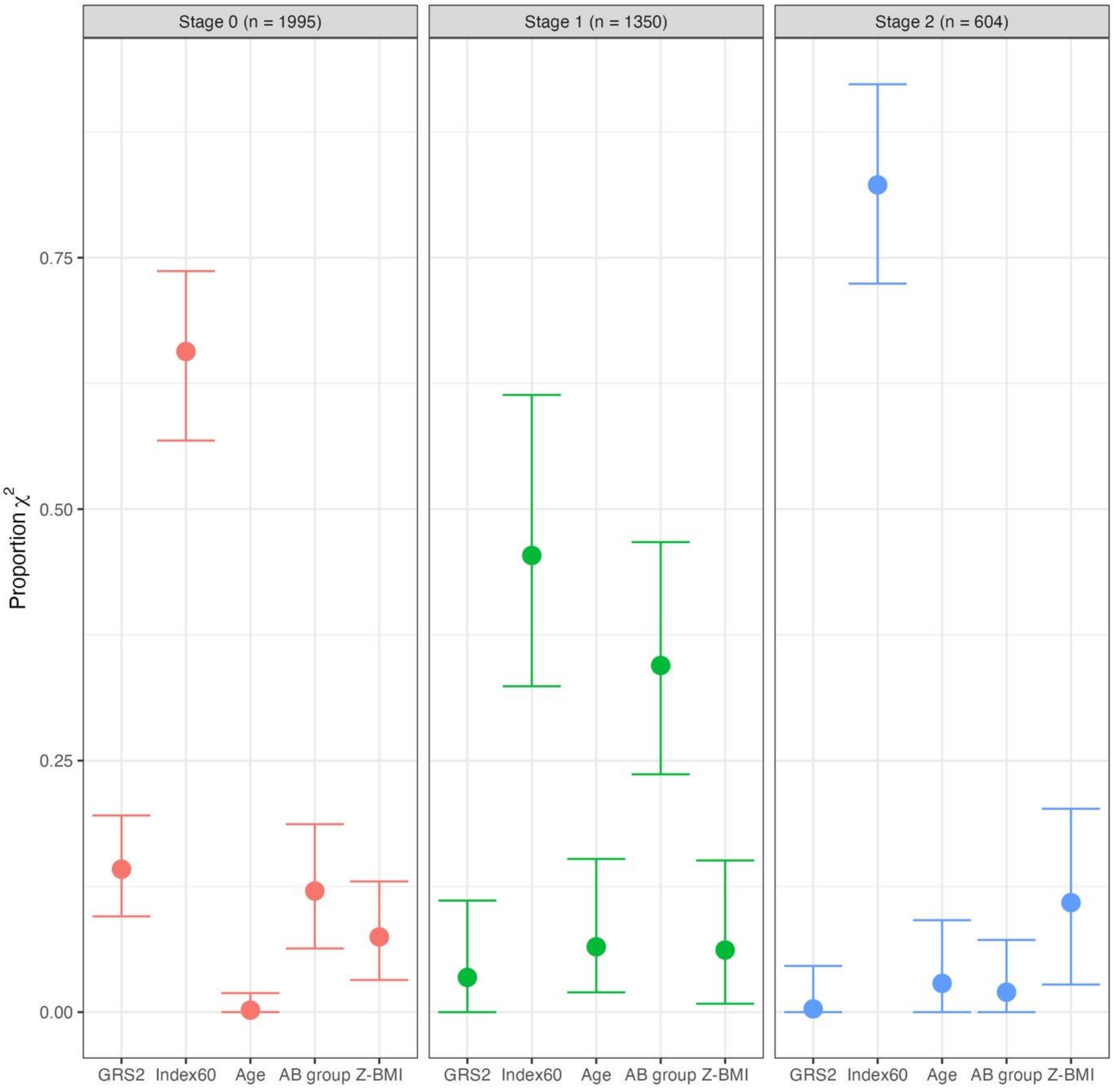

ESM Figure 3: Variable importance by type 1 diabetes stages in a model including GRS2, $Index_{60}$, age, autoantibody group and z-BMI. $Index_{60}$ is consistently the most important variable while GRS2 is only important at Stage 0 (i.e., single autoantibody positivity).

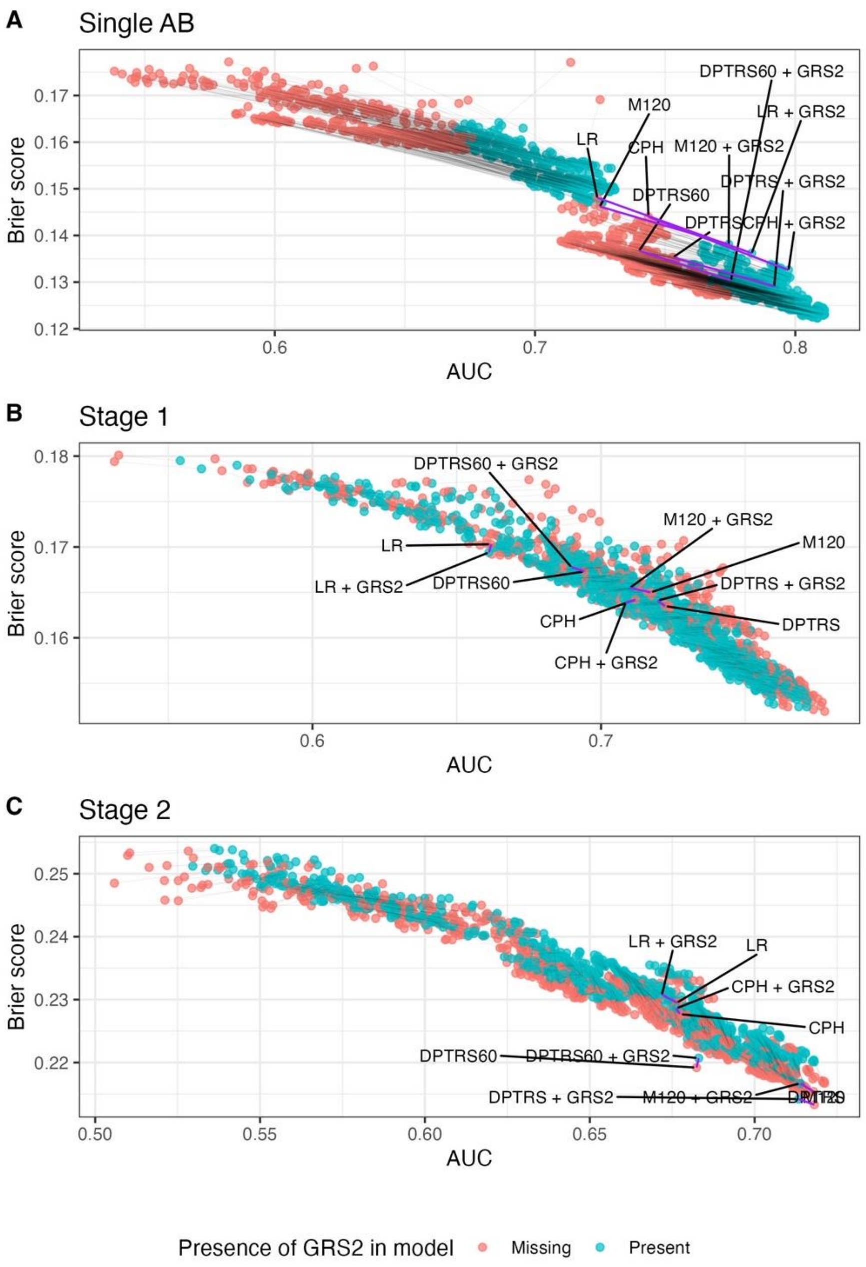

ESM Figure 4: Comparison of model performance with and without GRS2 including in the model formula when comparing time-dependent ROC AUC and Brier score at a 3-year horizon. At stage 0, there is generally a clear improvement by including GRS2 into the model. This effect can be observed for the classic models too. At stages 1 and 2, there is little to no improvement by including GRS2 into the model.

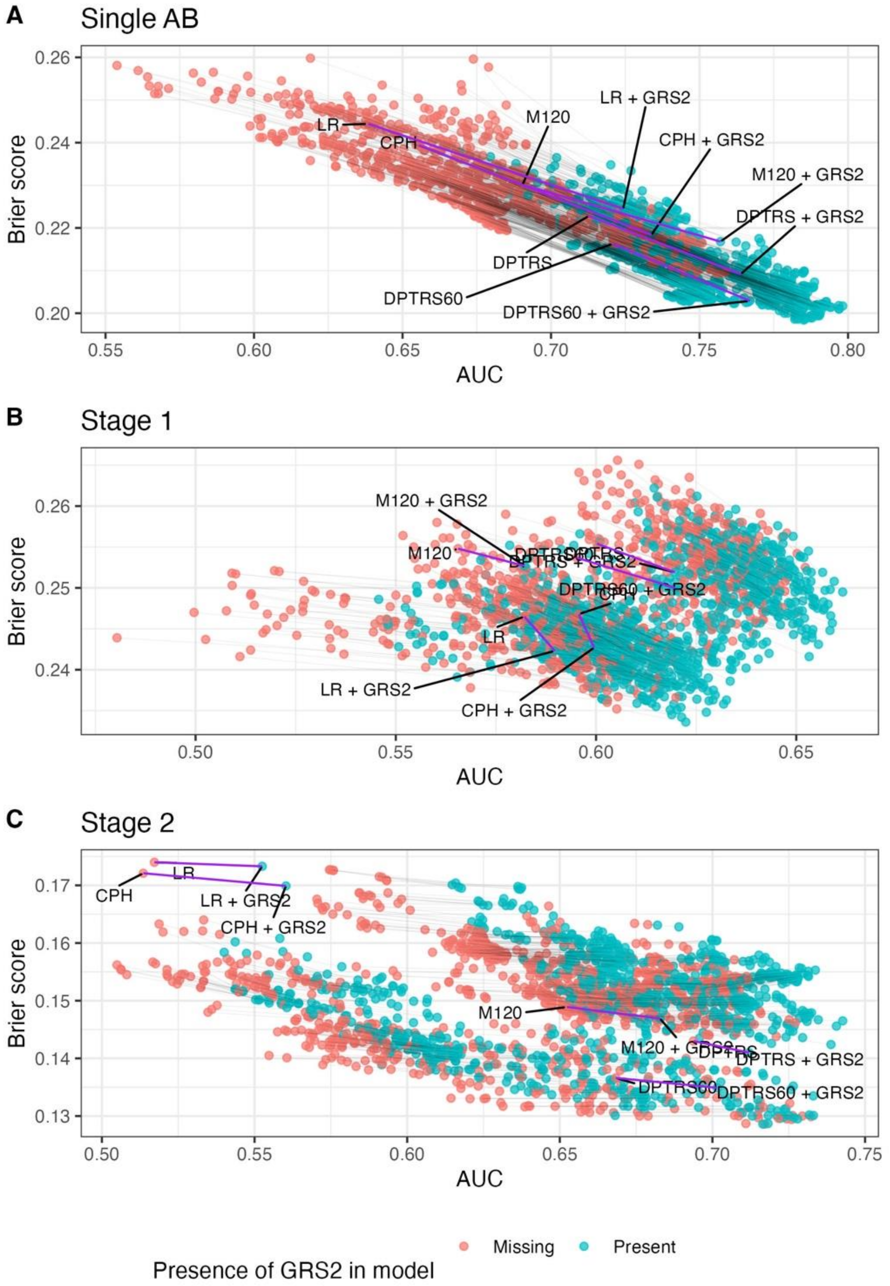

ESM Figure 5: Comparison of model performance with and without GRS2 including into the model when comparing time dependent ROC AUC and Brier score at 7 a year-horizon. At stage 0, there is generally a clear improvement by including GRS2 into the model. This effect can be observed for the classic models too. At stages 1 and 2, there is little to no improvement by including GRS2 into the model.

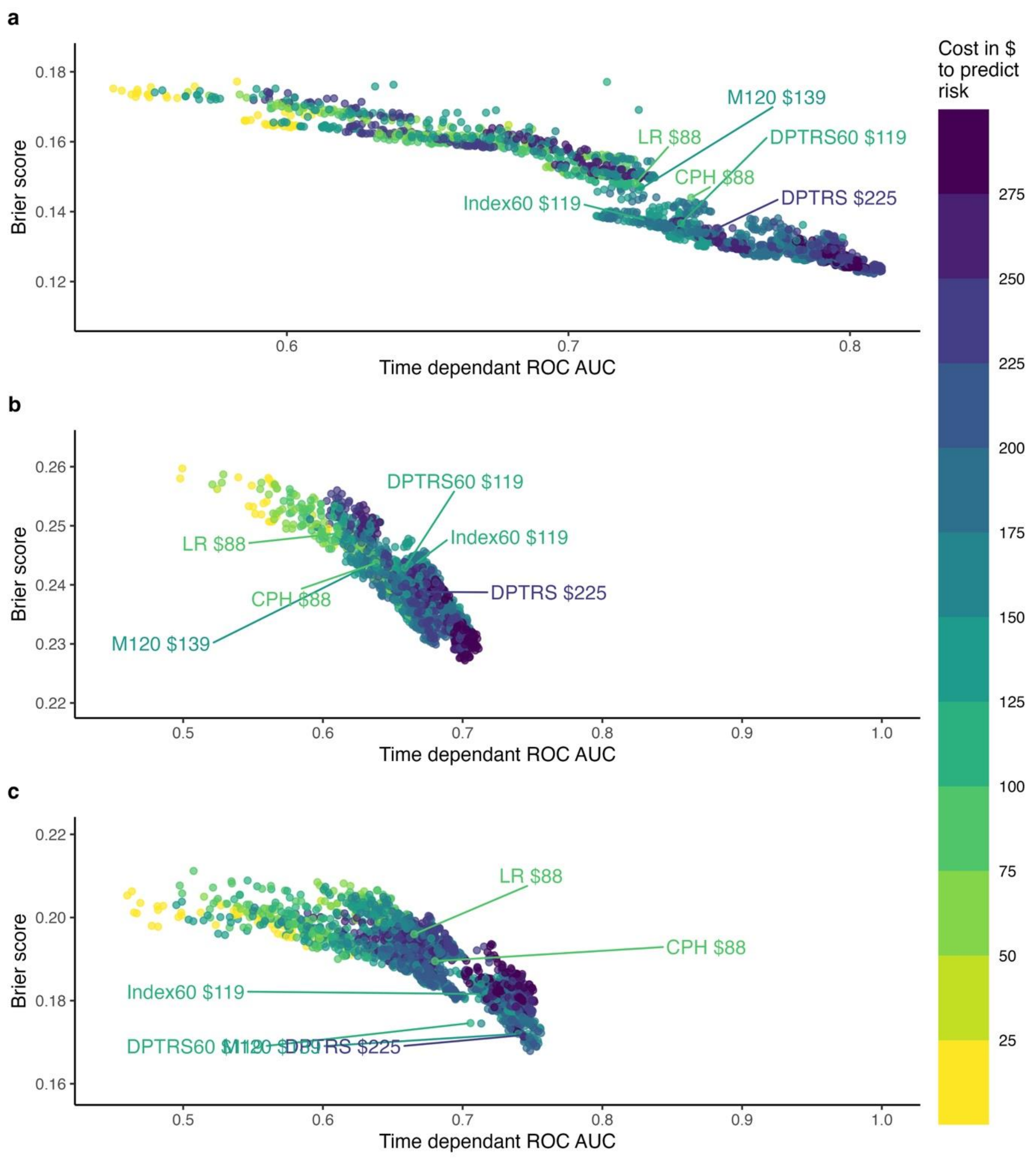

ESM Figure 6: Time-dependent ROC AUC and Brier score at a 5-year horizon. Each dot represents a Cox proportional hazard model with a different formula. The color scale indicates the cost of each model. Classic models previously described in the literature are labelled.

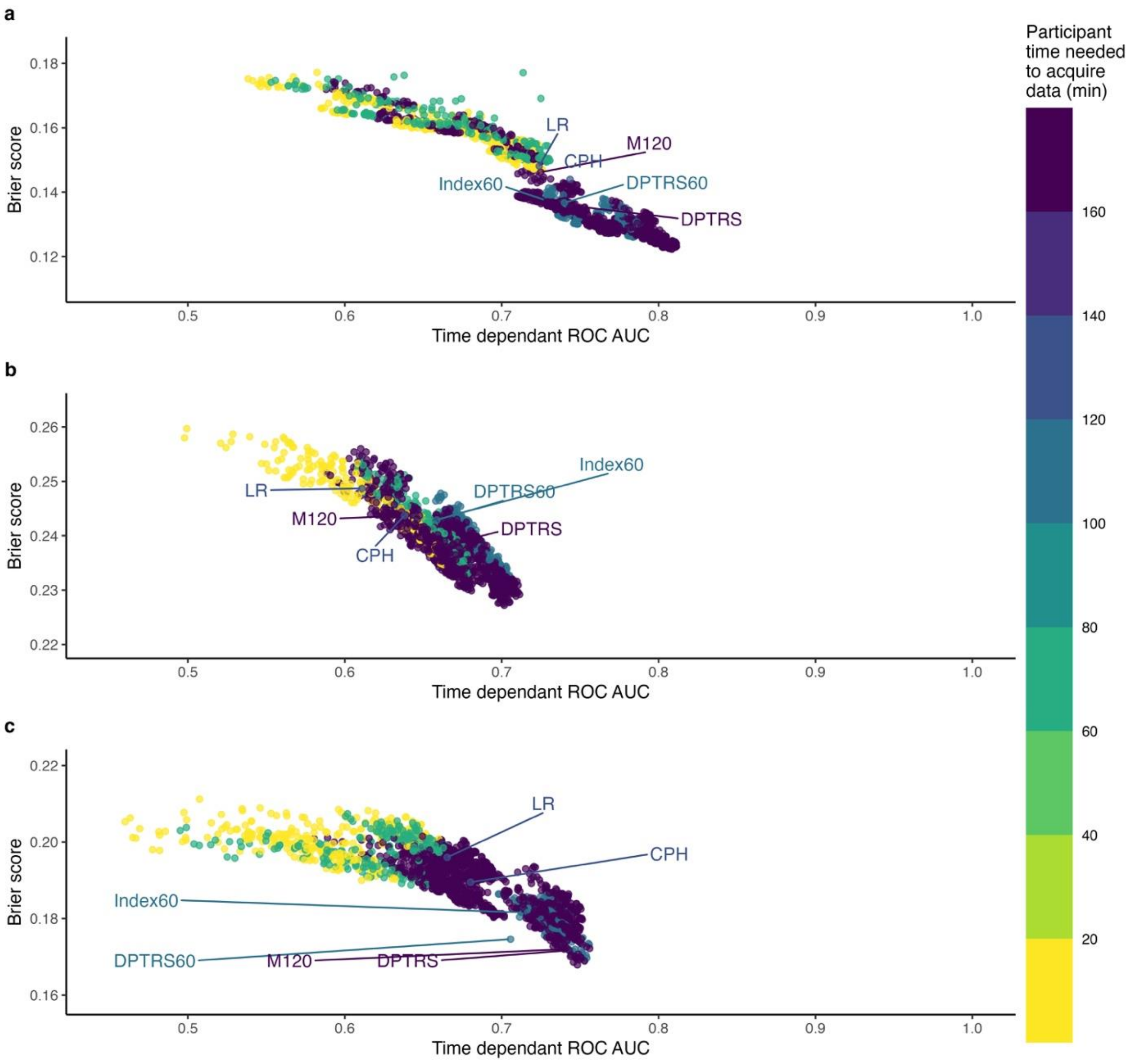

ESM Figure 7: Time-dependent ROC AUC and Brier score at a 5-year horizon. Each dot represents a Cox proportional hazard model with a different formula. The color scale indicates the patient time needed to inform each model. Classic models previously described in the literature are labelled.

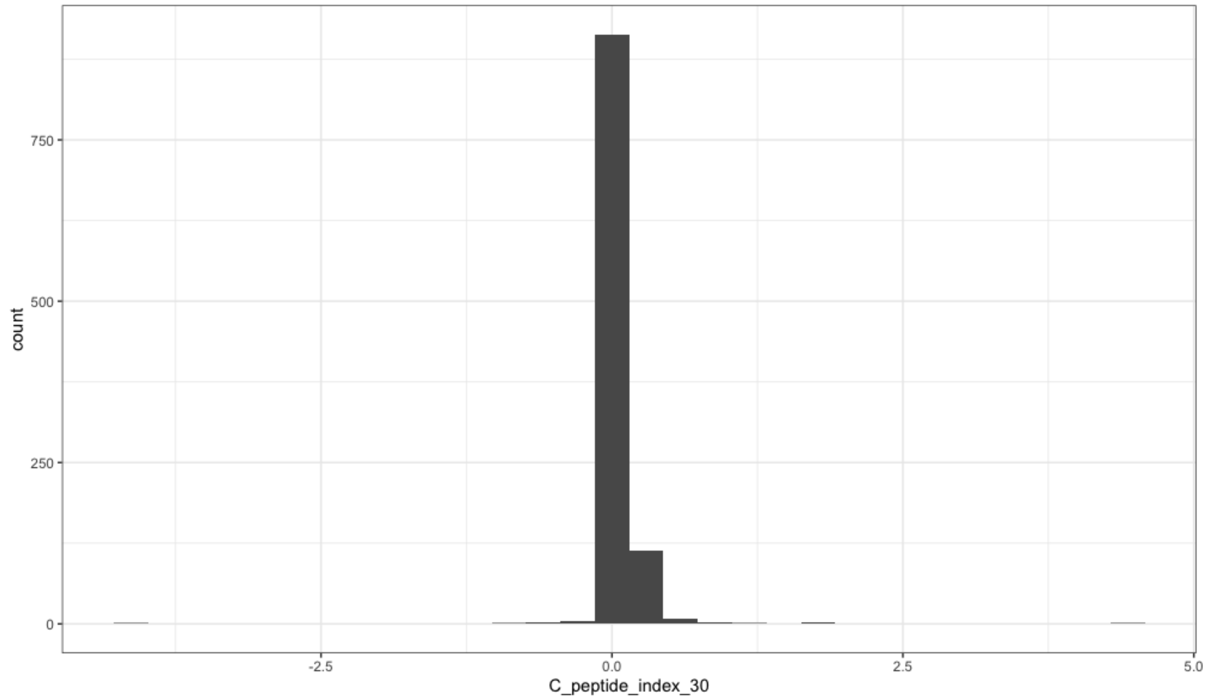

ESM Figure 8: histogram of the C-peptide$_{30}$ Index, one can observe the presence of extreme values which decrease the performance of the Cox proportional hazard model.

Type 1 Diabetes TrialNet Study Group

Personnel as of 4/28/2023.

Steering Committee: Kevan Herold (Yale University), Mark Anderson (University of California, San Francisco), Mark A. Atkinson (University of Florida), Todd Brusko (University of Florida), Jane Buckner (Benaroya Research Institute), Mark Clements (The Children's Mercy Hospital), Peter G. Colman (Walter & Eliza Hall Institute of Medical Research), Mark Daniels (Children's Hospital of Orange County), Linda DiMeglio (Indiana University), Carmella Evans-Molina (Indiana University), Jason Gaglia (Joslin Diabetes Center), Stephen E. Gitelman (University of California, San Francisco), Robin Goland (Columbia University), Peter Gottlieb (Barbara Davis Center for Childhood Diabetes), Michael Haller (University of Florida), Carla J. Greenbaum (Benaroya Research Institute), Martin Hessner (Medical College of Wisconsin), Jeffrey P. Krischer (University of South Florida), Megan Levings (University of British Columbia), Ingrid Libman (University of Pittsburgh), Peter Linsley (Benaroya Research Institute), Alice Long (Benaroya Research Institute), Sandra Lord (Benaroya Research Institute), Wayne Moore (The Children's Mercy Hospital), Antoinette Moran (University of Minnesota), Andrew Muir (Emory Children's Center), Priya Pralahad (Stanford University), William Russell (Vanderbilt Eskind Diabetes Clinic), Jennifer Sherr (Yale University), Lisa Spain (National Institute of Diabetes and Digestive and Kidney Diseases [NIDDK]), Andrea Steck (Barbara Davis Center for Childhood Diabetes), John Wentworth (Walter & Eliza Hall Institute of Medical Research), Diane Wherrett (University of Toronto), Perrin White (University of Texas Southwestern), Darrell M. Wilson (Stanford University), William Winter (University of Florida).
Past Members: Peter Antinozzi (Wake Forest University), David A. Baidal (University of Miami), Manuela Battaglia (San Raffaele University), Dorothy Becker (University of Pittsburgh), Penelope Bingley (University of Bristol), Emanuele Bosi (San Raffaele University), Richard Insel (JDRF), Thomas Kay (St. Vincent's Institute of Medical Research), Mikael Knip (University of Helsinki), Åke Lernmark (Skane University Hospital), Yuk-Fun Liu (University of Bristol), Jennifer B. Marks (University of Miami), Jerry Palmer (University of Washington), Mark Peakman (King's College), Louis Philipson (University of Chicago), Alberto Pugliese (University of Miami), Philip Raskin (University of Texas Southwestern), Maria Redondo (Baylor College of Medicine), Henry Rodriguez (University of South Florida Diabetes and Endocrinology Center), Bart Roep (Leiden University Medical Center), Desmond A. Schatz (University of Florida), Jay S. Skyler (University of Miami), Jay M. Sosenko (University of Miami), Jorma Toppari (Hospital District of Southwest Finland), Anette Ziegler (Technical University Munich).

Executive Committee: Kevan Herold (Yale University), Linda DiMeglio (Indiana University), Carla J. Greenbaum (Benaroya Research Institute), Jeffrey P. Krischer (University of South Florida), Ellen Leschek (National Institute of Diabetes and Digestive and Kidney Diseases [NIDDK]), Lisa Spain (National Institute of Diabetes and Digestive and Kidney Diseases [NIDDK]).
Past Members: Katarzyna Bourcier (National Institute of Allergy and Infectious Diseases [NIAID]), Richard Insel (JDRF), John Ridge (National Institute of Allergy and Infectious Disease [NIAID]), Jay S. Skyler (University of Miami).

Chair's Office: Kevan Herold, (Yale University), Lisa Rafkin (University of Miami).
Past Members: Carla J. Greenbaum (Benaroya Research Institute), Irene Santiago (University of Miami), Jay S. Skyler (University of Miami), Jay M. Sosenko (University of Miami).

TrialNet Coordinating Center (University of South Florida): Jeffrey P. Krischer, Brian Bundy, Michael Abbondondolo, Rajesh Adusumalli, Logan Alford, Matthew Boonstra, Jessica Conaty, David Cuthbertson, Julie Ford, Jennifer Garmeson, Veena Gowda, Cameron Hainline, Brian Hays, Kathleen Heyman, Christina Karges, Amy Kunz, Shu Liu, Kristin Maddox, Colleen Maguire, Margaret Moore, Sarah Muller, Melissa Murray, Johanna Nesbitt, Ryan O'Donnell, Melissa Parker, MJ Pereyra, Francisco Perez Laras, Aswani Raheja, Devon Rizzo, Ariana Rojas, Cintia Reichert, Lisa Steward, Michael Taylor, Roy Tamura, Dena Tewey, Elon Walker-Veras, Jianmei Wang, Melissa Wroble, Lili Wurmser, Lu You, Kenneth Young.
Past Members: Timothy Adams, Darlene Amado, Ilma Asif, Jenna Bjellquist, Laura Bocchino, Cristina Burroughs, Mario Cleves, Meagan DeSalvatore, Christopher Eberhard, Steve Fiske, Susan Geyer, Courtney Henderson, Martha Henry, Belinda Hsiao, Amanda Kinderman, Beata-Gabriela Koziol, Lindsay Lane, Ashley Leinbach, Jennifer Lloyd, Jamie Malloy, Julie Martin, Cameron McNeill, Jessica Miller, Thuy Nguyen, Jodie Nunez, Nichole Reed, Amy Roberts, Kelly Sadler, Tina Stavros, Christine Sullivan, Megan V. Warnock, Keith Wood, Rebecca Wood, Ping Xu, Vanessa Yanek.

National Institute of Diabetes and Digestive and Kidney Diseases [NIDDK]: Ellen Leschek, Lisa Spain.

Data Safety and Monitoring Board: Emily Blumberg (University of Pennsylvania), Sean Aas (Georgetown University), Gerald Beck (Cleveland Clinic Foundation), Rose Gubitosi-Klug (Case Western Reserve University), Dennis Wallace (Retired).
Past Members: David Brillon (Cornell University), Lori Laffel (Joslin Diabetes Center), Robert Veatch (Georgetown University), Robert Vigersky (Medtronic).

Infectious Disease Safety Committee: Brett Loechelt (Children's National Medical Center), Lindsey Baden (Brigham and Women's Hospital), Peter Gottlieb (Barbara Davis Center for Childhood Diabetes), Michael Green (University of Pittsburgh), Ellen Leschek (National Institute of Diabetes and Digestive and Kidney Diseases [NIDDK]), Ingrid Libman (University of Pittsburgh), Adriana Weinberg (University of Colorado), John Wentworth (Walter & Eliza Hall Institute of Medical Research).
Past Members: Nora Bryant (Joslin Diabetes Center), Yuk-Fun Liu (University of Bristol).

Laboratory Directors: Michael Sheldon (Infinity BiologiX), Adriana Weinberg (University of Colorado), William Winter (University of Florida), Liping Yu (Barbara Davis Center for Childhood Diabetes).
Past Members: Santica Marcovina (University of Washington), Jerry P. Palmer (University of Washington), Jay Tischfield (Rutgers University).

Psychosocial Committee: Suzanne Bennett-Johnson (Florida State University), Ryan McDonough (Children's Mercy), Kelli Delallo (University of Pittsburgh), Krim Driscoll (University of Florida), Kevan Herold (Yale University), Christine March

(University of Pittsburgh), Sarah McGaugh (Hospital for Sick Children Toronto), Arielle Pagryzinski (HUB), Lisa Rafkin (University of Miami), Jennifer Sherr (Yale University), Korey Hood (Stanford University), Diane Naranjo (Stanford University), Anna Barash (Benaroya), Holly O'Donnell (Barbara Davis Center), Brittany Bruggeman (University of Florida), Paige Trojanowski (Barbara Davis Center), Linda DiMeglio (Indiana University), Kali Johnson (University of Minnesota).

TrialNet Clinical Network Hub (Benaroya Research Institute): Arielle Pagryzinski, Emily Batts, Danielle Tom, Catherine Nguyen, Chris Budy.
Past Members: Annie Schultz, Kristin Fitzpatrick, Randy Guerra, Melita Romasco, Annie Shultz, Mary Ramey, Michele Patience-Staal, Meghan Tobin, Diana Skye, Christopher Webb.

Underrepresented Minority Committee: Ananta Addala (Stanford University), Susanne Cabrera (University of California San Francisco), Kevan Herold (Yale University, Lisa Rafkin (University of Miami), Andy Muir (Emory University), Robin Goland (Columbia University), Darrell Wilson (Stanford University), Ingrid Libman (University of Pittsburgh), Wayne Moore (The Children's Mercy Hospital), Carla Greenbaum (Benaroya), Linda DiMeglio (Indiana University), Brittany Bruggeman (University of Florida).

Active Personnel at Clinical Centers Participating in the TN01 Protocol:

Barbara Davis Center for Childhood Diabetes, Aurora, Colorado: Andrea K. Steck, Lexie Chesshir, Peter A. Gottlieb, Lisa Meyers, Aaron W. Michels, Marian Rewers, Kimber Simmons, Morgan Sooy, Fatima Tensun, Taylor Triolo, Leah Galvez Valencia, Paula Wadwa, Ruthie Williamson.

Benaroya Research Institute, Seattle, Washington: Carla J. Greenbaum, Jane H. Buckner, Sadiq El'Amin-White, Sandra Lord, Bao Ng, Mary Ramey, Michael Richter, Elaine Sachter, Corinna Tordillos, Dana VanBuecken, Kimberly Varner, Heather White, Nancy Wickstrom, Cassandra Williams, Alyssa Ylescupidez.

The Children's Hospital of Orange County: Amrit Bhangoo, Mark Daniels, Daina Dreimane, Mark Daniels, Marissa Erickson, Timothy Flannery, Nikta Forghani, Sarah Hu, Himala Kashmiri, Anabel Palencia, Christina Reh, Francoise Sutton, Heather Speer, Lien Trihn.

The Children's Mercy Hospital, Kansas City, Missouri: Wayne Moore, Fadi Al Muhaisen, Jennifer Boyd, Julia Broussard, Mark Clements, Aliza Elrod, Katelyn Evans, Max Feldt, Kelsee Halpin, Heather Harding, Jennifer James, Terri Luetjen, Ryan McDonough, Susan Mitchell, Tiffany Musick, Emily Paprocki, Rhiannon Pomerantz, Nikita Raje, Luis Sainz y Diaz, Britaney Spartz.

Columbia University, New York, New York: Robin Goland, Mone Anzai, Magdalena Bogun, Rachelle Gandica, Natasha Leibel, Jacqueline Lonier, James Pring, Nathan Schwab, Kristen Williams.

Emory Children's Center, Atlanta, Georgia: Andrew Muir, Amber Antich, Kristina Cossen, Eric Felner, Lynette Gonzalez, Wanda Sanchez, Catherine Simpson.

The Hospital for Sick Children, Toronto, Ontario: Diane K. Wherrett, Lesley Eisel, Sarah McGaugh, Rebecca Stochinsky, Mary Jo Ricci.

Indiana University, Indianapolis, Indiana: Linda A. DiMeglio, Carmella Evans-Molina, Jamie Felton, Heba M. Ismail, Megan Kirchner, Anna Neyman, Juan Sanchez, Corinne Parks-Schenck, Emily K. Sims, Maria Spall.

Stanford University, Stanford, California: Darrell M. Wilson, Bonnie Baker, Karen Barahona, Bruce Buckingham, Priya Prahalad, Trudy Esrey.

University of British Columbia, Kelowna, British Columbia: Constadina Panagiotopoulos, Daniel Metzger, Lauren Semkow.

University of California, San Francisco, California: Stephen Gitelman, Fatema Abdulhussein, Natalie Aceves, Mark Anderson, Julissa Cabrera, Hannah Chessner, Abby Cobb-Walch, Laura Dapkus, Aristides Diamant, Marysol Gonzales Granados, Tina Hu, Karen Ko, Janet Lee, Roger Long, Isabella Niu, Srinath Sanda, Caroline Schulmeister, Priya Srivastava, Lorraine Stiehl, Christine Torok, Rebecca Wesch, Jenise Wong, Kevin Yen.

University of Florida, Gainesville, Florida: Michael Haller, Mark Atkinson, Brittany Bruggeman, Todd M. Brusko, Miriam Cintron, Kristin Dayton, Timothy Foster, Jennifer Hosford, Laura Jacobsen, Sarah Peeling, Danielle Poulton, Desmond Schatz, Malinda Tran, William Winter.

University of Minnesota, Minneapolis, Minnesota: Antoinette Moran, Shannon Beasley, Melena Bellin, Jane Kennedy, Janice Leschyshyn, Brandon Nathan, Beth Pappenfus, Ihsan Rizky, Muna Sunni.

University of Pittsburgh, Pittsburgh, Pennsylvania: Ingrid Libman, Dorothy Becker, Kelli DeLallo, Christine March, Carly Shelleby, Frederico Toledo.

University of Texas Southwestern, Dallas, Texas: Perrin White, Abha Choudhary, Yasmin Dominguez, Philip Raskin, Serey Sao.

Vanderbilt Eskind Diabetes Clinic, Nashville, Tennessee: William Russell, Faith Brendle, Justin Gregory, Brenna Hammel, Jenny Leshko, Daniel Moore, Kimberly Rainer, Tyler Smith.

Walter and Eliza Hall Institute of Medical Research, Parkville, Victoria: Peter Colman, John M. Wentworth, Candice Breen, Marika Bjorasen, Spiros Fourlanos, Leonard Harrison, Felicity Healy, Leanne Redl.

Yale University, New Haven, Connecticut: Jennifer Sherr, Kevan Herold, Lori Carria, Jeanine May, William Tamborlane, Eileen Tichy, Stuart Weinzimer, Kate Weyman.
